# Multimodal Brain Graph Analysis for Uncovering Path‐Based Biomarkers in Schizophrenia: Integrating Static and Dynamic Approaches

**DOI:** 10.1002/hbm.70654

**Published:** 2026-10-21

**Authors:** Haleh Falakshahi, Hooman Rokham, Daniel H. Mathalon, Judith M. Ford, Bryon A. Mueller, Adrian Preda, Theo G. M. van Erp, Jessica A. Turner, Godfrey Pearlson, Vince D. Calhoun

**Affiliations:** ^1^ School of Electrical and Computer Engineering, Georgia Institute of Technology Atlanta Georgia USA; ^2^ Tri‐Institutional Center for Translational Research in Neuroimaging and Data Science (TReNDS), Georgia State University, Georgia Institute of Technology, and Emory University Atlanta Georgia USA; ^3^ Department of Psychiatry and Behavioral Sciences University of California, San Francisco San Francisco California USA; ^4^ San Francisco VA Medical Center San Francisco California USA; ^5^ Department of Psychiatry and Behavioral Sciences University of Minnesota Minneapolis Minnesota USA; ^6^ Department of Psychiatry and Human Behavior University of California, Irvine Irvine California USA; ^7^ Department of Psychiatry and Behavioral Health The Ohio State University Wexner Medical Center Columbus Ohio USA; ^8^ Department of Psychiatry Yale University School of Medicine New Haven Connecticut USA; ^9^ Olin Neuropsychiatry Research Center, Hartford Hospital Hartford Connecticut USA; ^10^ Department of Psychology Georgia State University Atlanta Georgia USA; ^11^ Department Computer Science Georgia State University Atlanta Georgia USA

## Abstract

Analyzing multimodal brain networks offers new insights into the mechanisms of brain disorders. However, most studies focus on unimodal networks or use global/local graph metrics, often missing critical information about disrupted pathways. In this study, we propose a novel framework for estimating static and dynamic multimodal brain graphs by integrating time‐resolved functional network connectivity (FNC) and gray matter features using a Gaussian graphical model. We then apply a path‐based graph theory analysis to identify connectors and disconnectors, edges that respectively introduce or eliminate paths in the patient graph compared to controls. Our findings in schizophrenia reveal several edges linked to missing or additional paths, indicating network disruptions that are undetectable using unimodal or pairwise analyses. These disrupted pathways span both within‐ and cross‐modal connections, particularly involving regions like the middle temporal gyrus and cerebellum. This work demonstrates the value of multimodal integration and emphasizes the importance of moving beyond individual edge‐level analysis when characterizing disease‐related network alterations. Our path‐centric approach provides a complementary view of brain connectivity disruption and identifies candidate path‐based markers of schizophrenia that warrant validation in independent datasets.

## Graphical abstract

A multimodal framework integrating time‐resolved fMRI connectivity and gray matter features enables estimation of static and dynamic brain graphs. Path‐based analysis identifies disrupted connectors and disconnectors in schizophrenia, revealing network alterations undetectable by unimodal or pairwise approaches and highlighting potential biomarkers of disease.

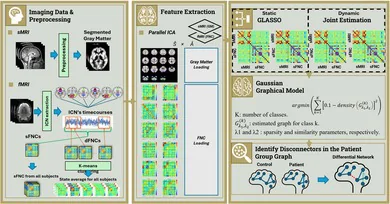

## 1 Introduction

Schizophrenia (SZ) represents a profound psychiatric disorder characterized by delusions and hallucinations, leading to a profound detachment from reality. Moreover, individuals often contend with flattened emotions, decreased pleasure, diminished motivation, restricted speech, social withdrawal, and cognitive impairments, rendering it a central focus of neuroimaging investigations (Rolls et al. 2020; Sass and Parnas 2003; Tost and Meyer‐Lindenberg 2012). Magnetic resonance imaging (MRI), a noninvasive technique, has been instrumental in uncovering the functional and structural mechanisms underlying SZ (Van Den Heuvel and Fornito 2004; Bora et al. 2011). Researchers have extensively documented abnormalities in brain structure and function with SZ, drawing upon data from structural magnetic resonance imaging (sMRI) and resting‐state functional magnetic resonance imaging (fMRI) to delineate the neural signatures associated with SZ.

Regarding structural brain abnormalities, individuals with SZ exhibit extensive cortical thinning, diminished cortical surface area, decreased gray matter (GM) volume, and reduced white matter (WM) volume, particularly prominent in frontal and temporal lobe regions (Chow et al. 2002; Lawrie and Abukmeil 1998; Van Erp et al. 2018). Functional brain abnormalities present as significant increases in regional homogeneity observed in crucial regions like the striatum, the right parahippocampal gyrus, and the right middle temporal gyrus, and individuals with SZ often exhibit functional connectivity abnormalities across a wide range of systems, commonly affecting prefrontal brain regions (Skaatun et al. 2017; van den Heuvel and Fornito 2014; Cole et al. 2011; Fornito et al. 2013). Therefore, our understanding of SZ has evolved to encompass the dysconnectivity hypothesis (Friston and Frith 1995), which posits that the disorder is characterized by structural and functional abnormalities in the brain, leading to disrupted connectivity between different brain regions. This hypothesis finds support in multimodal MRI studies (Calhoun et al. 2006; Skudlarski et al. 2010; Sugranyes et al. 2012), which have revealed widespread structural and functional dysconnectivity across various brain networks in individuals with SZ. The rationale behind integrating modalities lies in their capacity to unveil hidden connections among a range of complementary observations and pinpoint significant variations. This approach facilitates the synthesis of diverse findings in brain imaging, thereby enhancing our understanding of complex neurological phenomena. However, despite the wealth of information they offer, the full potential of multimodal MRI studies in SZ remains largely untapped due to the specialized expertise required for their comprehensive analysis and interpretation, distinguishing them from unimodal studies.

Recent advancements in network science and graph theory have further enriched our ability to analyze the topological relationships among brain regions (Reijneveld et al. 2007; Stam and Reijneveld 2007). Brain graph analysis is widely utilized to identify potential biomarkers for mental illnesses like SZ. In structural brain graph studies, metrics like path length and global efficiency have demonstrated disruptions in SZ (v. d. Heuvel et al. 2010; Yu et al. 2018). In functional brain graph analyses of SZ, findings revealed reduced pairwise functional connectivity alongside increased variability. Moreover, graph metrics such as degree, clustering coefficient, global efficiency, and local efficiency exhibited reductions, while path length demonstrated an increase in individuals with SZ (Yu et al. 2018; Bassett et al. 2012; Liu et al. 2008). With growing interest in multimodal analysis of brain imaging data, an array of approaches is emerging to construct multimodal brain graphs (Yu et al. 2018; Wang et al. 2010; Van den Heuvel 2013; Liang 2013; Tewarie et al. 2014). These methods offer valuable insights into the physiological, electrophysiological, and genetic underpinnings of brain connectivity's topological properties. Despite advancements in comprehending multimodal brain graphs in SZ, significant gaps persist within the field. First, prevailing studies primarily employ static connectivity measures, overlooking the dynamic alterations inherent in brain networks, which are known to be affected in SZ. Second, there exists a scarcity of research directly comparing multimodal brain graphs between control and patient cohorts, impeding the identification of specific alterations linked to the disorder. Third, previous multimodal investigations often analyze data from distinct modalities separately and then correlate the independent findings or utilize one modality to constrain models corresponding to another. While valuable, such approaches underutilize cross‐modality information, which can be better leveraged through data fusion methodologies (Calhoun and Sui 2016; Abrol et al. 2017). Feature‐based data fusion techniques, for example, extract relevant features from each modality independently and subsequently assess the relationships between these features. By capitalizing on the partial information available in each modality, this approach unveils data variations not evident through unimodal analyses (Calhoun and Sui 2016). Finally, prior brain graph analyses have predominantly relied on Pearson correlation, overlooking the influence of other variables (He 2019). Despite its common usage, Pearson's correlation fails to distinguish whether two brain components are directly or indirectly connected through another brain component. Furthermore, existing literature has primarily focused on standard graph metrics like clustering coefficient and average path length to identify biomarkers, often overlooking the intricate details of disrupted pathways within brain graphs when comparing control and patient groups.

Our study presents a pioneering method for estimating multimodal graphs, applicable to both static and dynamic scenarios, and subsequently identifying path‐based biomarkers that differentiate between control and patient groups. Our innovation primarily lies in our methodology. Unlike previous studies, which often treated data from different modalities independently or employed one modality to constrain models corresponding to another, we employ data fusion techniques (Calhoun and Sui 2016). Specifically, we utilize parallel independent component analysis (pICA) as our data fusion algorithm. pICA is a hypothesis‐free statistical technique that extends ICA to simultaneously analyze two modalities, identifying independent components and estimating their interrelationships (Liu and Calhoun 2007). Moreover, we enhance the complexity of our brain graph framework by integrating functional network connectivity (FNC) and gray matter characteristics as nodes. This comprehensive approach fills a notable gap in current research, providing a deeper understanding of brain connectivity patterns. Additionally, we analyze these multimodal graphs in both static and dynamic contexts, shedding light on the temporal dynamics of brain connectivity. In terms of modeling, we depart from conventional techniques by employing Gaussian graphical models (GGMs) based on partial correlation to represent the brain graph. This departure from Pearson correlation‐based approaches allows for a more nuanced and accurate depiction of the underlying connectivity structure. We utilize the GGM framework to analyze paths between control and patient groups in multimodal brain graphs derived from resting‐state fMRI data and structural MRI (sMRI). GGM, widely used for investigating relationships among multiple random variables, serves here to elucidate interactions between brain components. Within a GGM, two variables are deemed conditionally independent given all other variables if their corresponding off‐diagonal element in the precision matrix (the inverse of the covariance matrix) is zero, indicating the absence of an edge between them. Thus, the precision matrix in GGM succinctly encapsulates the conditional dependence within the network structure (He 2019). Finally, building on previous research on path analysis (Falakshahi et al. 2022), we aim to identify edges indicative of missing or additional paths within the patient group graph. We hypothesize that disparities between control and patient groups stem from disruptions in multilink pathways. Our ultimate goal is to uncover multimodal path‐based biomarkers for individuals with SZ using a graph theory approach, both in static and dynamic contexts.

The rest of the paper is organized as follows: The Materials and Methods section elucidates our approach, encompassing the estimation of static and time‐varying multimodal brain graphs for both control and patient groups, as well as our path analysis approach. Results delineate the outcomes of our path analysis conducted on static and dynamic multimodal brain graphs from individuals with SZ and control groups. The Discussion section examines our findings and their implications. Finally, we offer concluding remarks in the Conclusion section.

## 2 Materials and Method

In this section, we outline our methodology for constructing static and dynamic multimodal brain graphs for both control and patient groups in a data‐driven fashion. Subsequently, we delve into path analysis conducted on the multimodal brain graphs, comparing patterns between control and patient cohorts. Figure 1 provides an overview of the materials and methods employed in this study.

**FIGURE 1:**
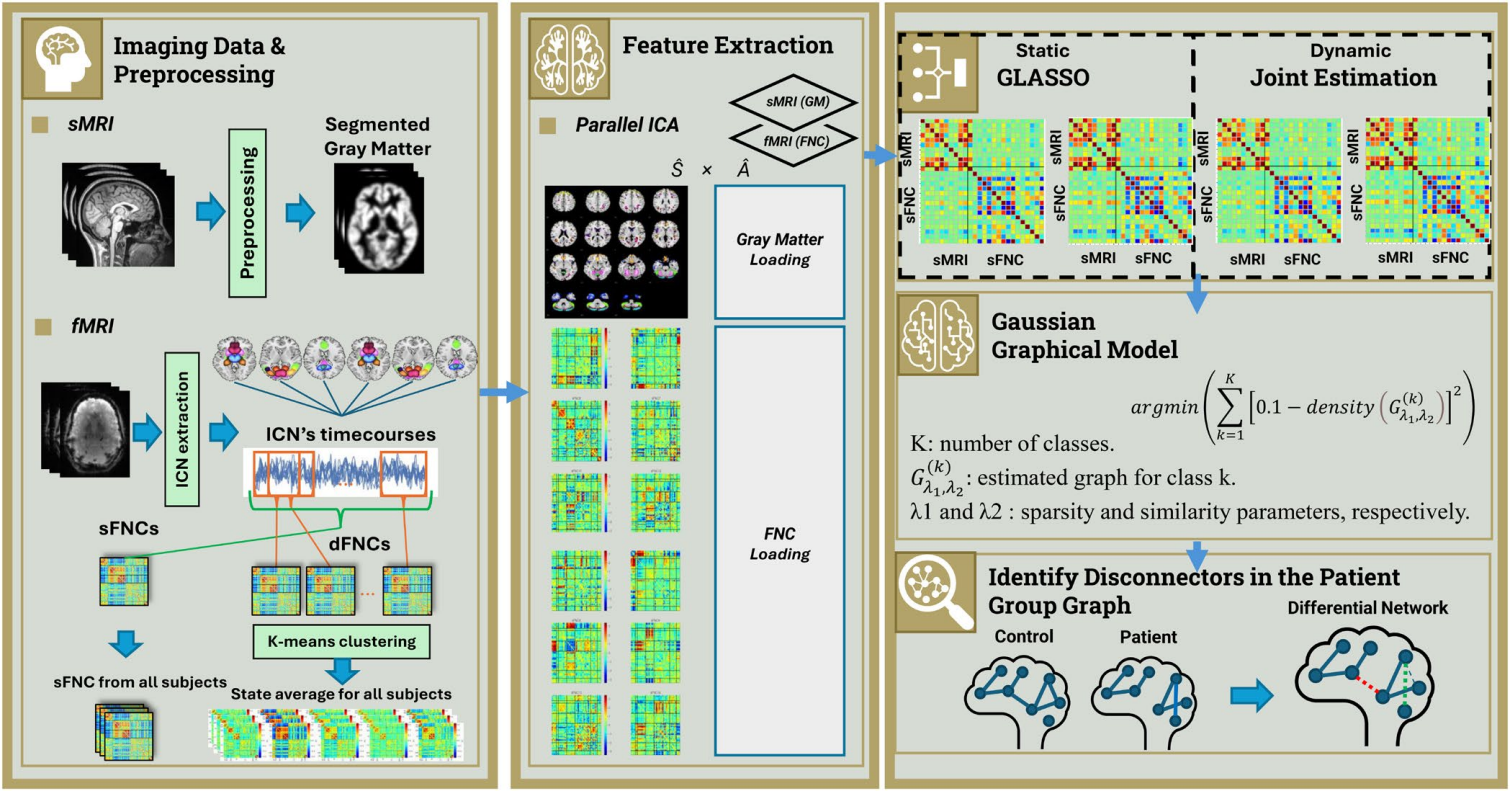
Overview of the static and dynamic multimodal graph‐analysis framework. Structural MRI and resting‐state fMRI data from 160 HC participants and 151 individuals with SZ were used to derive gray matter (GM) and functional network connectivity features. For the static analysis, static FNC and GM features were fused using parallel ICA (pICA), and the resulting subject‐level loading coefficients were adjusted for acquisition site, age, and sex and pooled‐standardized across all participants. Separate graphical lasso models were estimated for the HC and SZ groups using a common regularization parameter selected to minimize deviation from a prespecified target graph density of 0.10. For the dynamic analysis, dynamic FNC was estimated using a sliding‐window approach, recurrent connectivity states were identified using k‐means clustering, and GM and state‐averaged dFNC features were fused using pICA. State‐specific precision matrices were estimated using joint graphical lasso. The resulting graph topologies were subjected to path‐based differential network analysis to identify disconnectors associated with loss of paths and connectors associated with additional paths in SZ relative to HC.

### 2.1 Data Information, Preprocessing, and Intrinsic Connectivity Network Extraction

We utilized data from the fBIRN study (Keator et al. 2016) comprising sMRI and fMRI scans collected from 160 controls and 151 SZ patients, with all subjects providing written informed consent. Demographic details from the fBIRN dataset are summarized in Table 1. sMRI scans underwent preprocessing using statistical parametric mapping (SPM12), available at http://www.fil.ion.ucl.ac.uk/spm/. This preprocessing involved unified segmentation and normalization of the scans into distinct gray matter, white matter, and cerebrospinal fluid (CSF) compartments. The segmentation employed a modulated normalization algorithm to compute gray matter, followed by smoothing with a Gaussian kernel (full width at half maximum (FWHM) of 6 mm) to enhance data quality. The fBIRN sample consisted of 160 healthy controls (HCs) and 151 individuals with schizophrenia (SZ). Participant inclusion criteria required head motion during resting‐state fMRI acquisition to remain within 3° of rotation and 3 mm of translation, as well as successful spatial normalization with near‐full brain coverage. As previously reported for this fBIRN sample, the HC and SZ groups were matched for age (*p* = 0.1758) and sex (*p* = 0.3912) (Fu et al. 2021).

**TABLE 1:** Demographics of the fBIRN cohort.

	Healthy control	Schizophrenia
Number	160	151
Age	37.0 ± 10.9	38.8 ± 11.6
Gender	45F^a^/115 M	36F/115 M
PANSS		
Positive	—	15.31 ± 4.96
Negative	—	14.30 ± 5.60
cMIND		
Speed of Processing	0.06 ± 1.02	−1.36 ± 1.09
Attention	0.03 ± 0.99	−1.36 ± 1.40
Working Memory	0.04 ± 0.97	−1.16 ± 1.08
Verbal Learning	0.04 ± 1.00	−1.38 ± 1.19
Visual Learning	0.03 ± 1.00	−1.09 ± 1.14
Reasoning Problem Solving	0.36 ± 0.98	−0.85 ± 1.22
cMIND composite	0.04 ± 0.95	−1.59 ± 1.22

^a^ F, female; M, male.

For fMRI data preprocessing, we employed the SPM12 toolbox. Initial 5 time points of the fMRI data were excluded to ensure stable magnetization. Rigid‐body motion correction and slice‐timing correction were applied to address motion‐related artifacts and temporal discrepancies, respectively. In addition to this preprocessing step, subjects exceeding 3° of rotational motion or 3 mm of translational motion were excluded according to the fBIRN quality‐control criteria. Subsequently, spatial normalization aligned the fMRI images with the standard Montreal Neurological Institute template using an echo‐planar imaging template, with slight resampling to achieve isotropic voxels measuring 3 × 3 × 3 mm^3^. To ensure compatibility with the sMRI data, smoothing with a Gaussian kernel (FWHM of 6 mm) was applied to the fMRI images. Next, a spatially constrained group‐independent component analysis approach within the Neuromark pipeline (Du et al. 2020) was applied to the functional data, yielding 53 consistent components corresponding to intrinsic connectivity networks (ICNs). Each ICN comprises a collection of voxels exhibiting robust synchronization of spontaneous fluctuations in the BOLD signal, effectively representing a cohesive functional unit.

Employing fully‐blind ICA may yield varying components identified across datasets. Such inconsistency in identified components could hinder replication or comparison efforts. To mitigate this challenge and ensure consistency in the identification of ICNs between control and SZ groups, we adopted the fully automated NeuroMark pipeline. This pipeline utilizes spatially constrained ICA informed by reliable network templates (Du et al. 2020). Here we used the NeuroMark_fMRI_1.0 tepmlate. To establish this template and ascertain robust and reproducible ICNs, spatial ICA was applied to two large datasets of typical controls: the Human Connectome Project (HCP, 823 subjects after subject selection) and the Genomics Superstruct Project (GSP, 1005 subjects after subject selection). Group ICA with a model order of 100 was independently performed on each dataset to derive group‐level components. Subsequently, independent components from the HCP and GSP datasets were matched based on spatial similarity (correlation ≥ 0.4), and reproducible ICNs were selected as the network templates. ICN identification and functional domain labeling were carried out by five neuroscience experts, with further details provided in (Du et al. 2020). NeuroMark provides a common spatial framework for identifying corresponding ICNs across participants and acquisition sites using reproducible network templates derived from independent datasets. This facilitates comparison of subject‐specific ICNs across sites and datasets but does not constitute an explicit statistical harmonization procedure for removing scanner or site related variability. No separate scanner harmonization procedure was applied in the present study. To account for potential site‐related variation in the static multimodal graph analysis, acquisition site was explicitly included as a categorical nuisance variable, together with age and sex, during adjustment of the subject‐level pICA loading coefficients prior to graph estimation. The treatment of these covariates in the static graph analysis is described in Section 2.2. Following the generation of Neuromark templates, spatial maps and time courses (TCs) for the fBIRN dataset were obtained using these templates as prior information within a spatially constrained ICA algorithm. For this algorithm, we employed multivariate objective optimization independent component analysis with reference (MOO‐ICAR), implemented in the GIFT toolbox from TReNDS (http://trendscenter.org/software/gift) (Iraji et al. 2020), as it has demonstrated good performance in our prior studies (Du and Fan 2013; Du et al. 2016). These 53 ICNs were further categorized into seven functional domains: auditory, cerebellar, cognitive‐control, default‐mode, sub‐cortical, sensorimotor, and visual.

The HC and SZ groups were matched for age and sex in the fBIRN cohort. Static and dynamic FNC group differences were evaluated using two‐sample *t*‐tests. For the static multimodal graph analysis, subject‐level pICA loading coefficients were additionally adjusted for acquisition site, age, and sex and subsequently standardized using the pooled mean and standard deviation across all 311 participants prior to graph estimation, as described in Section 2.2. The same standardization transformation was therefore applied to both diagnostic groups. Head motion was addressed through rigid‐body motion correction and predefined exclusion criteria of 3° rotation and 3 mm translation; a subject‐level summary measure of residual motion was not included as an additional covariate. Medication exposure, illness duration, and cognitive performance were not included as covariates in the statistical models.

### 2.2 Analysis of Static Multimodal Brain Graphs in Control Versus Patient Groups

To construct a multimodal brain graph incorporating both sFNC and GM features, it is crucial to establish a framework for defining nodes and edges across modalities. This entails employing pICA as a data fusion technique to identify independent components and estimate their relationships across modalities. We utilized pICA, implemented in the FIT toolbox from TReNDS (http://trendscenter.org/software/fit), to fuse sFNC and GM maps as input features. The optimal number of components was determined using the elbow criterion based on explained variance, resulting in 15 components. Selection of model order is an important consideration in pICA. Previous simulation‐based evaluation of the pICA methodology demonstrated generally stable component estimation across varying signal‐to‐noise ratios and cross‐modal connection strengths, while also showing that substantial underestimation or overestimation of component number can affect estimation of cross‐modal relationships (Liu and Calhoun 2007). Therefore, rather than specifying the model order arbitrarily, we used a data‐driven elbow criterion to identify an appropriate decomposition order. Following pICA decomposition, component maps were visually inspected for anatomical interpretability and potential artifactual or noise‐like spatial patterns, and 4 GM components were excluded from subsequent static analyses. pICA produces four matrices: two loading matrices, each corresponding to a modality, with dimensions (number of subjects: 311) × (number of components: 15), and two‐component matrices with dimensions (number of components: 15) × (number of GM voxels/sFNC measures).

Following pICA decomposition, we estimated static multimodal Gaussian graphical models separately for the HC and SZ groups. Prior to graph estimation, each retained subject‐level pICA loading coefficient was adjusted for acquisition site, age, and sex while retaining diagnosis in the regression model. Acquisition site was represented as a categorical fixed effect. Because the structural and functional pICA loading coefficients differed in numerical scale, the adjusted coefficients were subsequently standardized using the pooled mean and standard deviation calculated across all 311 participants. The same standardization transformation was therefore applied to both the HC and SZ groups.

Separate graphical lasso models (Friedman et al. 2008) were then estimated for the HC and SZ groups. A common regularization parameter was applied to both diagnostic groups to avoid introducing group differences through differential regularization. The regularization parameter was selected using a prespecified target‐density criterion. Specifically, candidate values of α were evaluated and the common α that minimized the summed squared deviation of the HC and SZ graph densities from a target density of 0.10 was selected. Graph density was calculated from the nonzero off‐diagonal elements of the estimated precision matrices. The selected value was subsequently used to estimate the final HC and SZ graphs using all participants.

Partial‐correlation matrices were derived from the corresponding precision matrices to characterize conditional dependencies among multimodal nodes. Edge‐wise *p*‐values and Benjamini‐Hochberg FDR‐adjusted values were additionally calculated to characterize inferential support for individual conditional associations.

Following the estimation of multimodal GGMs for both control and patient groups, we proceeded to compare and analyze the paths between multimodal nodes in these groups. Drawing on previous research in path analysis (Falakshahi et al. 2022), we focused on identifying specific scenarios within the patient group graph compared to the control group. In particular, we examined instances where there existed at least one path between two nodes in the control group but not in the patient group, signifying a disconnection between them. Such edges in the patient group, causing the absence of paths, were termed “disconnectors.” Conversely, we explored cases where no path existed between two nodes in the control group, but there was at least one in the patient group, indicating the presence of additional paths. These edges in the patient group, facilitating new connections, were termed “connectors.” Essentially, disconnectors led to disconnection, while connectors fostered abnormal integration within the patient group graph. To address this analysis, we employed the concept of connected components in graph theory. Connected components ensure that every node can be reached from every other node through a series of edges, known as paths (Balakrishnan and Ranganathan 2012). In a brain graph based on GGM, if two nodes belong to the same connected component, they are conditionally dependent given all other nodes.

By considering the path structure of the control group graph and utilizing the concept of connected components, we identified disconnectors associated with absent paths and connectors associated with additional paths in the patient group graph. Initially, we identified all connected components in the control graph. If any of these components fragmented into multiple connected components in the patient group, we determined the missing edges causing disconnectivity (disconnectors) by mimicking the path structure of the control group. Additionally, if any connected components in the patient group contained multiple connected components from the control group, we identified additional new edges (connectors) by referencing the structure of paths in the control group graph. It is worth noting that not all missing edges in the patient group lead to disconnection, and similarly, not all additional edges trigger new connections in the patient group. Figure 2 provides a detailed illustration of the process of identifying disconnectors and connectors.

**FIGURE 2:**
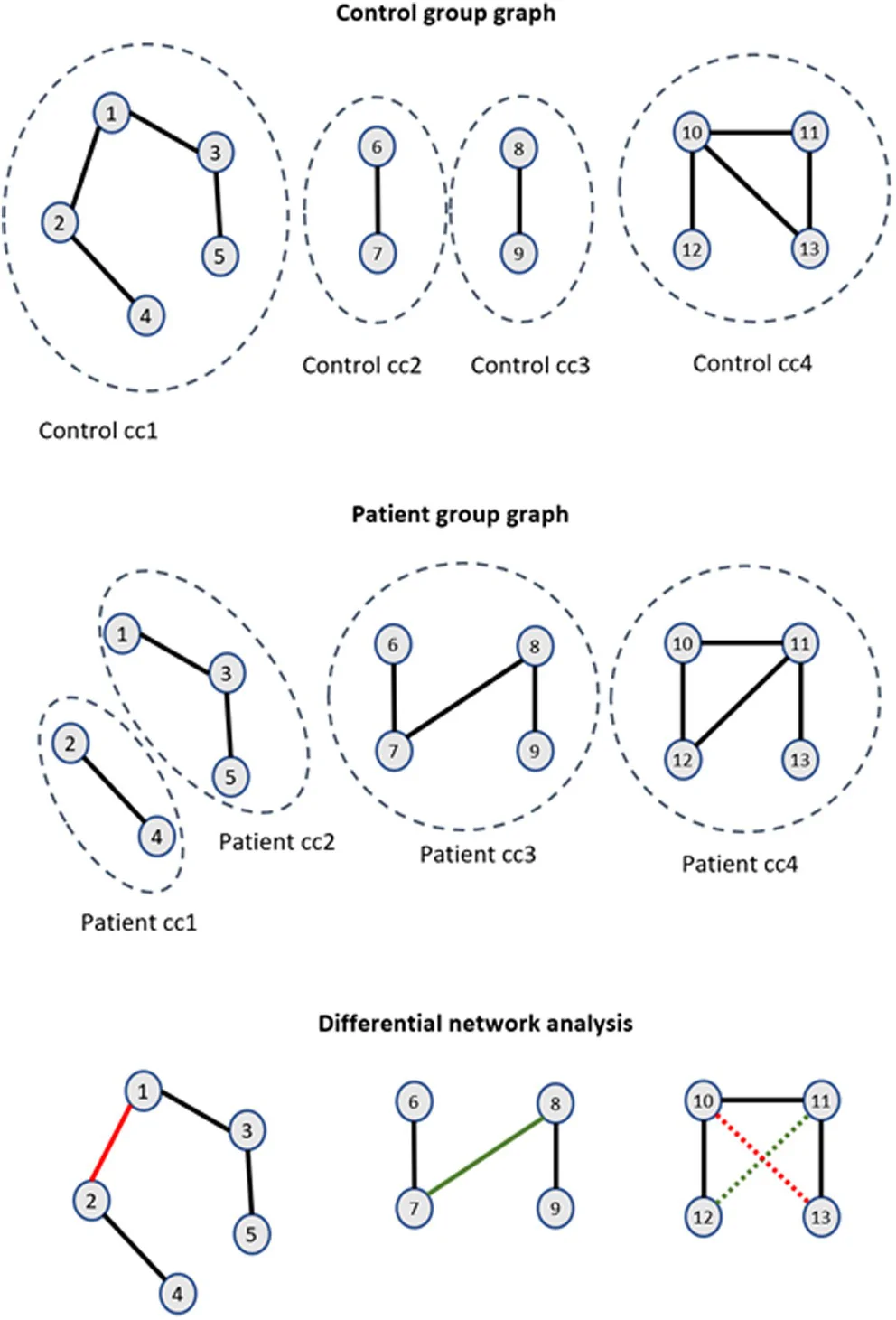
The top row illustrates an example of the control group graph, showcasing four distinct connected components labeled as Control cc1, Control cc2, Control cc3, and Control cc4. In contrast, the second row depicts an example of the patient group graph, exhibiting a different configuration compared to the control group, also comprising four connected components. Discrepancies between the patient and control group graphs manifest as both missing and additional edges within the patient group graph. The objective is to identify edges within the patient group graph that either lead to disconnection or foster abnormal integration, by replicating the graph structure observed in the control group. For instance, the nodes {1, 2, 3, 4, 5} form a connected component in the control group graph under “Control cc1,” where each node is reachable from the others. However, in the patient group graph, these nodes are split into two disconnected connected components, “Patient cc1” and “Patient cc2,” with no path between them. Consequently, edge (1,2), highlighted in red in the third row, is deemed a disconnector, as it causes this disconnection. Conversely, nodes {6, 7, 8, 9}, which in the control group graph are part of separate connected components, become consolidated into a single connected component labeled “Patient cc3” in the patient group graph. Notably, edge (7, 8), marked in green in the third row, is identified as a connector, as it facilitates this abnormal integration within the patient group graph. It is important to note that not all missing edges contribute to disconnection, nor do all new additional edges necessarily result in abnormal integration. For instance, nodes {10, 11, 12, 13}, forming “Control cc4,” remain within a single connected component labeled “Patient cc4” in the patient group graph. Although edge (10, 13) is absent in the patient group graph, it doesn't disrupt connectivity as these nodes remain reachable within a single connected component. Similarly, while edge (11, 12) is a new addition in the patient group graph, it doesn't establish a new connection compared to the control group graph. Simple missing and additional edges are depicted with dashed red and green lines, respectively, in the third row.

### 2.3 Analysis of Dynamic Multimodal Brain Graphs in Control Versus Patient Groups

Initially, we embarked on computations for dynamic functional network connectivity (dFNC), a method delving into the variations in interactions between distinct brain networks over time. By analyzing dFNC, we gain insight into the temporal changes in FNCs within ICNs. For each participant, we estimated dFNC using a sliding window approach, a technique that allows us to observe how connectivity evolves over time. We employed tapered windows in this sliding window technique to investigate the dynamic connectivity among ICNs. Specifically, the time courses (TCs) of ICNs were divided into segments using a sliding window consisting of 20 time points with a step size of 1 time point, resulting in 137 windows in total. Estimating FNC among these segmented ICN TCs was facilitated using the GIFT toolbox (http://trendscenter.org/software/gift/). Given the potential lack of comprehensive data in shorter time segments for capturing covariance matrices, we utilized a regularized precision matrix approach for covariance estimation. This involved employing the graphical LASSO method (Friedman et al. 2008) with L1 norm regularization to encourage sparsity, applied to the windowed data. The regularization parameter (*λ*) was determined through a cross‐validation framework, ensuring robustness in the covariance estimation process.

To investigate recurrent dFNC patterns, we applied k‐means clustering to the windowed FNC data and grouped them into distinct clusters using L1 norm as the distance metric. Subject‐specific exemplars were identified as those time windows exhibiting local maxima in dFNC variance across all pairs. Subsequently, k‐means clustering was performed on these exemplars, iterated 100 times with random initialization of centroid positions, to establish functional states, represented by group cluster centroids. These centroids served as starting points for clustering the windowed dFNC data across all subjects. The optimal number of states was determined using the elbow criterion, resulting in five clusters, which align well with the established range observed in prior dFNC studies across various brain disorders (Allen et al. 2014; Tu et al. 2019; Fu et al. 2018; Fiorenzato et al. 2019). In evaluating differences between control and patient groups, we conducted univariate tests in dFNC analysis. Initially, we computed the mean for each state across subjects, derived from the subject‐specific FNC windows corresponding to that state, representing a characteristic connectivity pattern for each subject‐state pair. To identify significant group differences, *t*‐tests were performed for each pair of ICNs based on the averaged dFNC values across states. Multiple comparison correction was applied using the FDR method, with a significance threshold set at 0.05.

To estimate dynamic multimodal graph, we applied pICA on GM and state average features, with state average FNCs all stacked, and replicated GM if the same subject was in multiple states. Similarly, the optimal model order for the dynamic multimodal analysis was determined using the elbow criterion based on explained variance, resulting in 15 GM components and 14 dFNC components. Following visual inspection, 2 GM components exhibiting predominantly artifactual or noise‐like spatial patterns were excluded from subsequent analyses. Following pICA decomposition, the loading matrices were unpacked separately for each dynamic state, resulting in state‐specific samples because not all participants contributed windows to every state. Relying on expert knowledge we removed two artifactual components of GM maps. Group‐specific precision matrices for each dynamic state were estimated using joint graphical lasso (JGL). The JGL formulation jointly estimates the precision matrices for the HC and SZ groups while incorporating sparsity and similarity penalties controlled by $\lambda_{1}$ and $\lambda_{2}$, respectively. To obtain sparse and interpretable state‐specific graphs, $\lambda_{1}$ and $\lambda_{2}$ were selected using grid search to minimize the deviation of the estimated graph densities from a target density of 0.10:

(1) $$\text{argmin}\left(\sum_{k=1}^{K}{\left[0.1-\text{density}\left(G_{\lambda_{1},\lambda_{2}}^{\left(k\right)}\right)\right]}^{2}\right)$$

where *K* is number of classes and $G_{\lambda_{1},\lambda_{2}}^{\left(k\right)}$ is corresponding estimated graph for group *k* and λ1 and λ2 are sparsity and similarity parameters, respectively. Partial‐correlation matrices were subsequently derived from the estimated precision matrices, statistically thresholded using FDR correction, and subjected to the connector/disconnector path analysis described above.

To assess the influence of unequal diagnostic‐group representation across dynamic states, we performed a repeated balanced‐sample sensitivity analysis. For each state, the group with the larger number of contributing participants was randomly downsampled without replacement to match the smaller group. The resulting balanced sample sizes were 78, 42, 98, 135, and 63 participants per group for States 1–5, respectively. The original pICA component definitions and state‐specific JGL regularization parameters were retained, and the state‐specific GGMs and connector/disconnector analyses were re‐estimated for 500 random balanced samples. Stability of each original path finding was quantified as the proportion of iterations in which that connector or disconnector was recovered.

## 3 Results

### 3.1 Static Analysis Results

Based on expert judgment, four artifactual components from the GM maps were eliminated. The plot depicting the remaining 11 GM components is displayed in Figure 3, while Figure 4 illustrates the sFNC maps arranged in a component‐by‐component matrix format (53 × 53).

**FIGURE 3:**
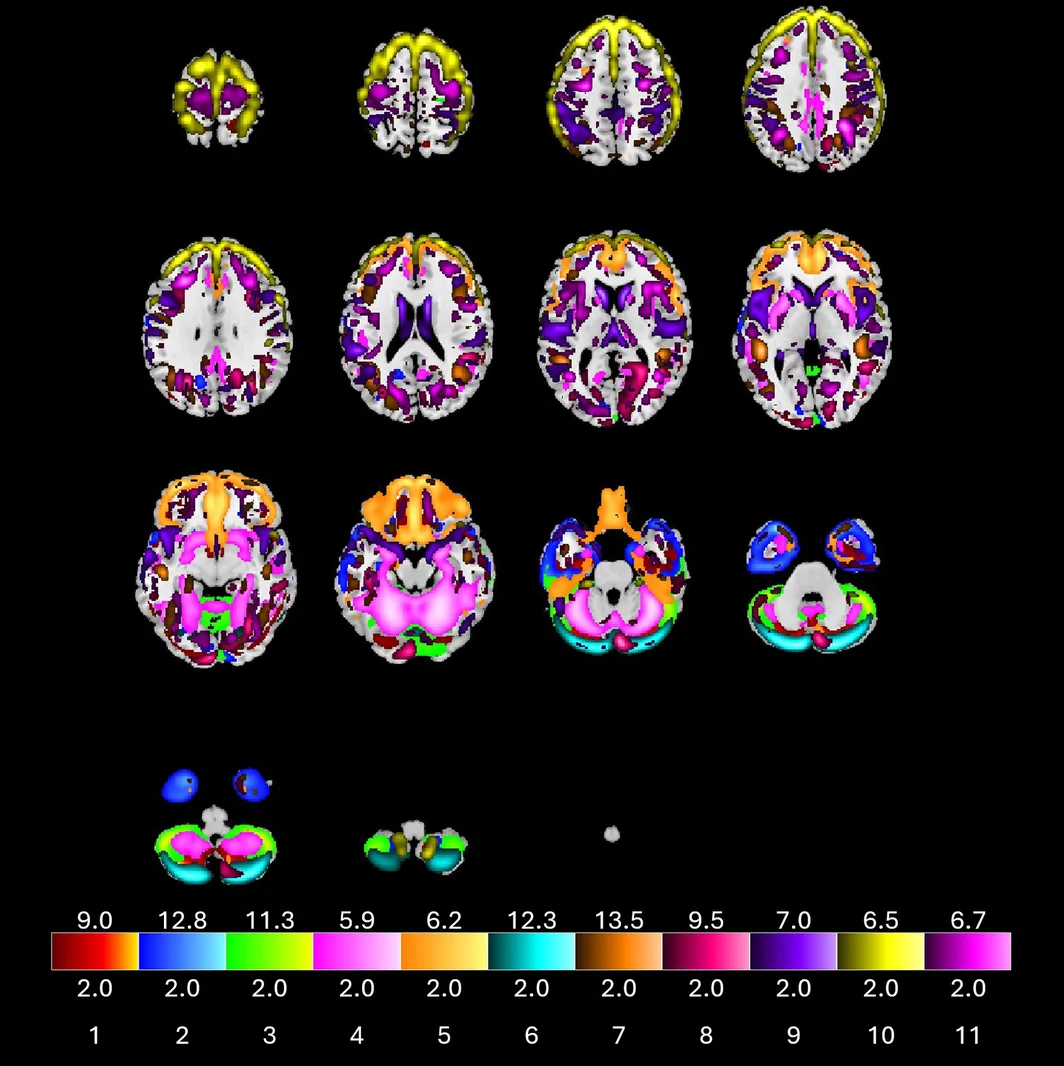
GM components estimated from pICA utilizing gray matter (GM) and static FNC as input data. Following the rigorous exclusion of artifacts and noisy components, a total of 11 robust GM components were identified through the pICA methodology. Components identified across different brain regions including cerebellum, calcarine, temporal middle gyrus, frontal middle gyrus.

**FIGURE 4:**
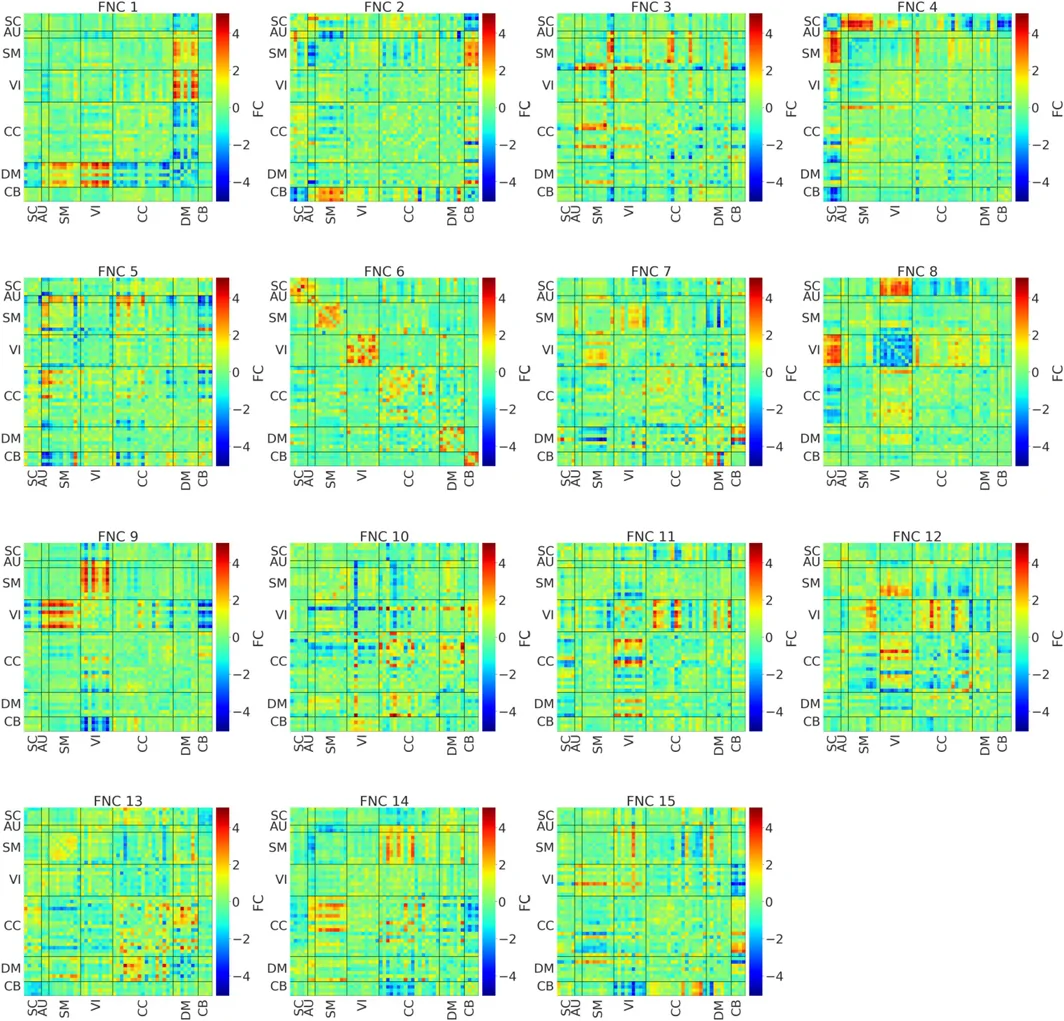
sFNC components. A total of 15 sFNC components were estimated using pICA and shown in component‐by‐component matrix form. Each component shows different connectivity patterns. For instance, FNC1 shows high activation between default mode networks and other brain regions, and FNC9 shows high activation between visual domain and other brain regions. (AU: Auditory, CB: Cerebellar, CC: Cognitive‐control, DM: Default‐mode, SC: Sub‐cortical, SM: Somatomotor, VI: Visual).

Univariate tests were performed on sFNC to evaluate group differences. The group‐specific mean sFNC matrices are shown in Figure 5 along with the group differences. Compared to the HC, the SZ group showed significantly weaker connectivity between all AUD, SM, and VIS networks. Additionally, the SZ group showed lower antagonist connectivity between SC networks and AUD, VIS, and SM networks. The connectivity between the cerebellar and subcortical ICNs is also higher in the control group.

**FIGURE 5:**
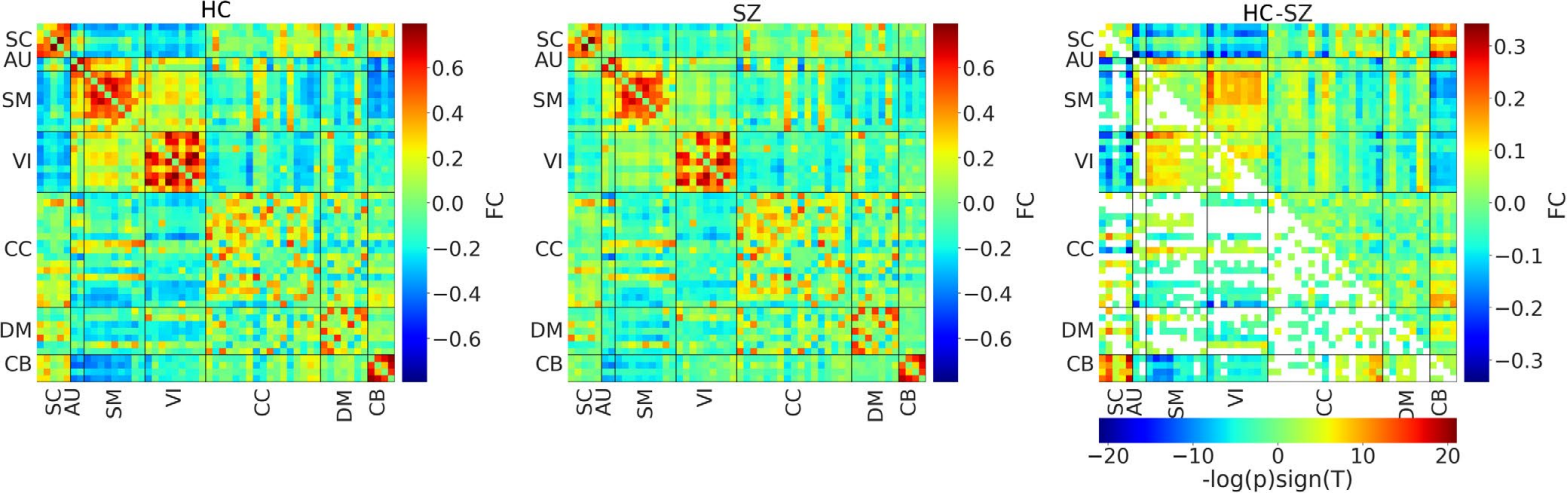
Mean sFNC maps for control (left) and individuals with SZ (middle) groups and the result of group differences (right), the upper triangle is the group difference (SZ‐HC) in sFNC, and the lower triangle is the results of multiple comparison test. The SZ group showed significantly weaker connectivity between all AUD, SM, and VIS networks. The SZ group exhibited reduced connectivity among the subcortical networks and the networks related to auditory (AUD), visual (VIS), and somatomotor (SM) functions. Additionally, the connectivity between the cerebellar and subcortical intrinsic connectivity networks (ICNs) was found to be elevated in the control group.

Using the target‐density criterion, a common graphical lasso regularization parameter of *α* = 0.3853 was selected for the static multimodal graph analysis. The resulting site‐, age‐, and sex‐adjusted and pooled‐standardized HC graph contained 34 edges, corresponding to a density of 0.1046, whereas the SZ graph contained 32 edges, corresponding to a density of 0.0985. Across the union of the two graphs, 26 edges were shared between HC and SZ, three were simple missing edges in SZ, and five were simple additional edges in SZ.

Path‐based differential network analysis identified five disconnectors in the SZ graph: (sMRI_10, sMRI_15), (sMRI_14, sFNC_14), (sFNC_6, sFNC_13), (sFNC_6, sFNC_14), and (sFNC_7, sFNC_14). One connector, (sFNC_5, sFNC_15), was identified as an edge associated with additional path integration in SZ relative to HC. Of the path‐altering edges, one disconnector (sMRI_14, sFNC_14) was cross‐modal, whereas the remaining disconnectors and the connector occurred within modality. Figure 6 illustrates the shared, simple missing, simple additional, connector, and disconnector edges in the final static graph analysis.

**FIGURE 6:**
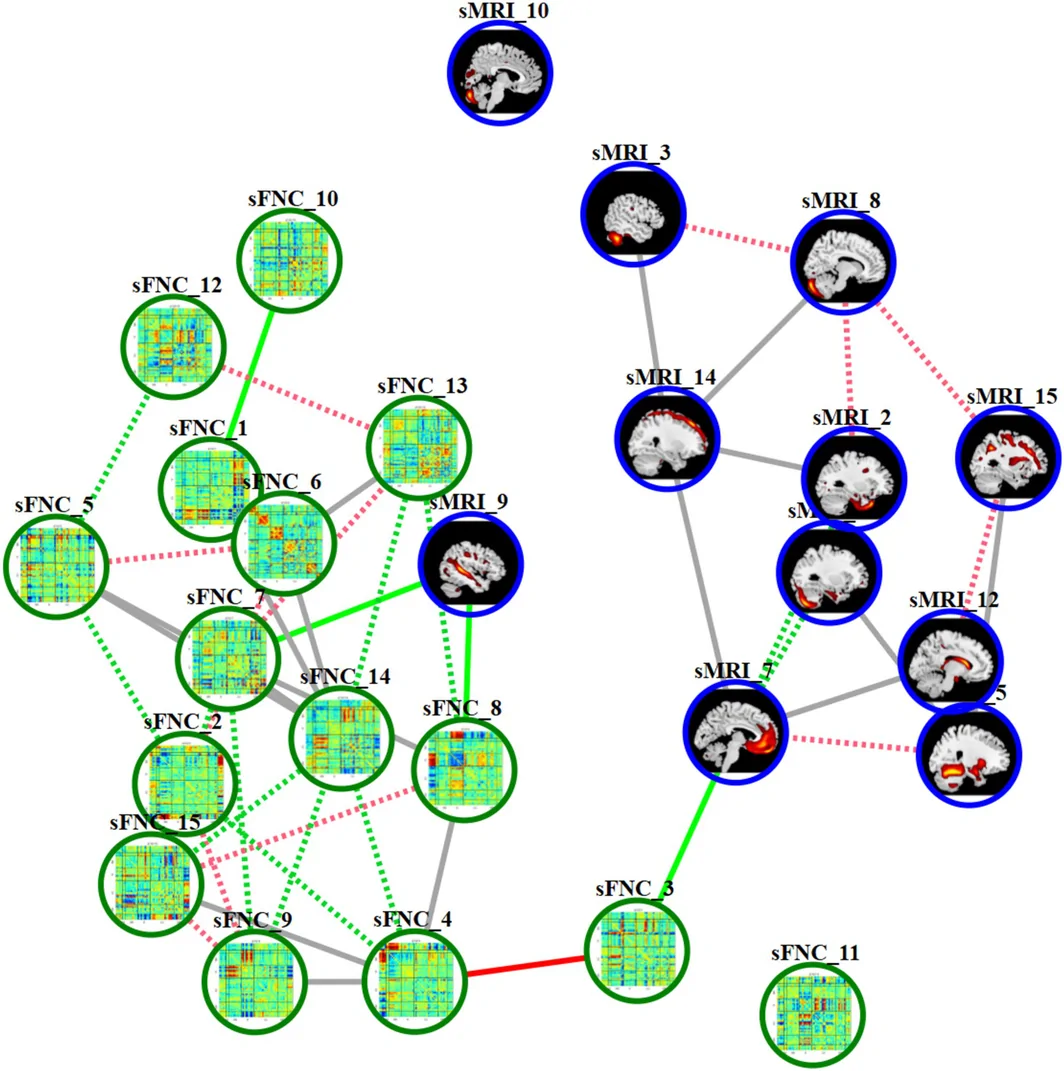
Static multimodal path‐analysis results. Group‐specific Gaussian graphical models were estimated using separate graphical lasso models for the HC and SZ groups after adjustment of the pICA loading coefficients for acquisition site, age, and sex and pooled standardization across all participants. A common regularization parameter was selected to minimize deviation of the two graph densities from a prespecified target density of 0.10. Gray solid edges indicate edges shared between the HC and SZ graphs. Dashed red and green edges indicate simple missing and additional edges in SZ, respectively, that do not alter path reachability. Solid red edges indicate disconnectors associated with loss of paths in SZ relative to HC, whereas solid green edges indicate connectors associated with additional path integration in SZ. The static analysis identified five disconnectors and one connector, including one cross‐modal disconnector (sMRI_14–sFNC_14).

### 3.2 Dynamic Analysis Results

After the removal of two artifacts, the GM components are visible in Figure 7. Figure 8 illustrates the dFNC maps arranged in a component‐by‐component matrix format (53 × 53). Additionally, the average functional connectivity for each state is depicted in Figure 9. State 3, 5, and 2 exhibit a moderate to high correlation pattern, while states 1 and 4 demonstrate low and weakest connectivity states. The group‐specific medians for each state are displayed in Figure 9 (top two rows). Notably, State 2 reveals the most pronounced correlation between the AUD, SM, VI, SC, and CB domains, alongside notable antagonism between SC and AU, SM, VI, as well as between AU, SM, VI, and CC, DM, CB. Similar patterns with varying degrees of intensity are observed in states 5 and 3. Conversely, State 1 and 4 depict weak connectivity between domains, and within State 4, connectivity patterns are solely observed within domains. In group difference evaluations, the number of significant FDR corrected *p* values were 173 for State 1, 55 for State 2, 41 for State 3, 57 for State 4, and 34 for State 5. Figure 9 also illustrates the results of the multiple comparison test for the control and SZ group. It is important to observe that not all subjects have dynamic windows that are assigned to every state, therefore the number of observations (subject‐specific matrices, and consequently the degrees of freedom in the model) changes with each state. Analyzing group differences among states reveals that the observed notable antagonism between the cerebellar and sensory ICNs, which exhibited reduced levels in patients with SZ, is evident within the control group during state 1. In state 1, the control group displayed the most pronounced hyperconnectivity between the subcortical and cerebellar domains. Furthermore, the patient groups exhibited elevated hyperconnectivity between the subcortical and cognitive control ICNs. Notably, state 1 showcased heightened connectivity between cognitive control and sensory ICNs in the control group. Notable differences, primarily centered around the subcortical domain and other domains, were identified in state 2. Additionally, certain distinctions were noted between cognitive control and cerebellar, as well as sensorimotor and cognitive domain ICNs. Similarly, states 3, 4, and 5 exhibited analogous differences, primarily associated with the subcortical domain and various other domains, with particular emphasis on the subcortical and cerebellar domains.

**FIGURE 7:**
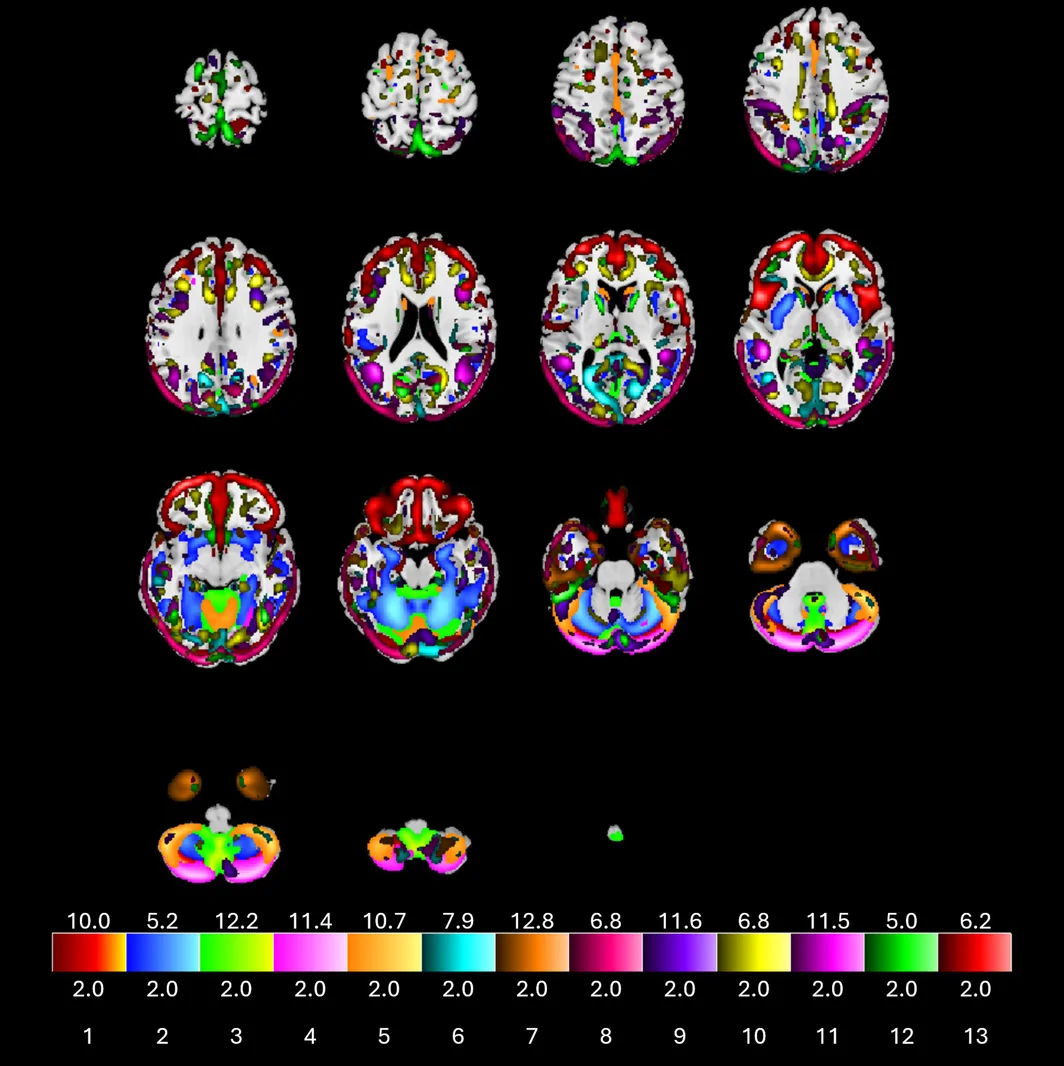
Plot of 13 GM components. The components were estimated from the pICA analysis for the dFNC analysis. The components highlight different brain regions including the cerebellum, calcarine, temporal pole, temporal mid, and frontal regions. These networks represent coherent patterns of brain structure that are consistent across individuals.

**FIGURE 8:**
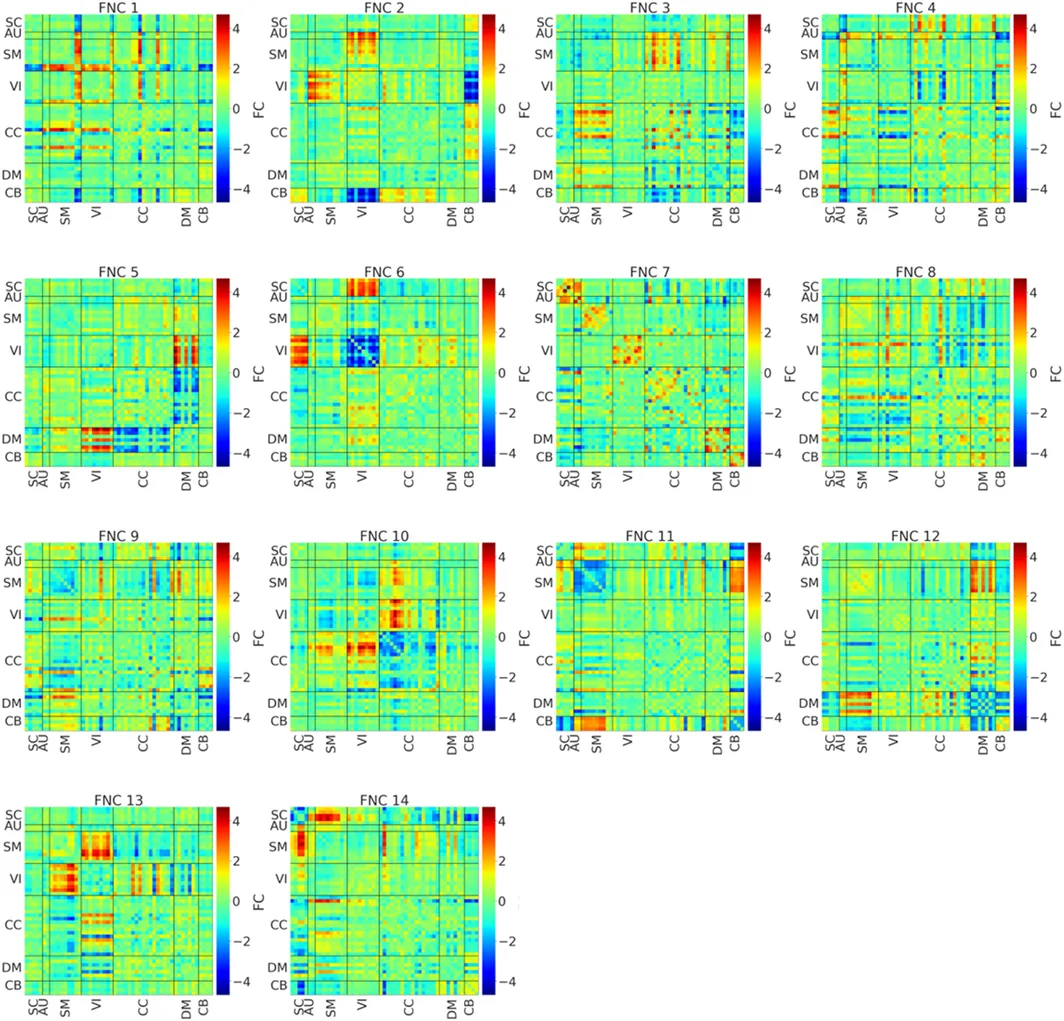
dFNC components were estimated using pICA in component‐by‐component matrix form. For instance, FNC5 shows high activation between default mode networks and other brain regions, and FNC6 shows high activation between visual domain and other brain regions. These components represent coherent patterns of functional connectivity that are consistent across individuals. (AU: Auditory, CB: Cerebellar, CC: Cognitive‐control, DM: Default‐mode, SC: Sub‐cortical, SM: Somatomotor, VI: Visual).

**FIGURE 9:**
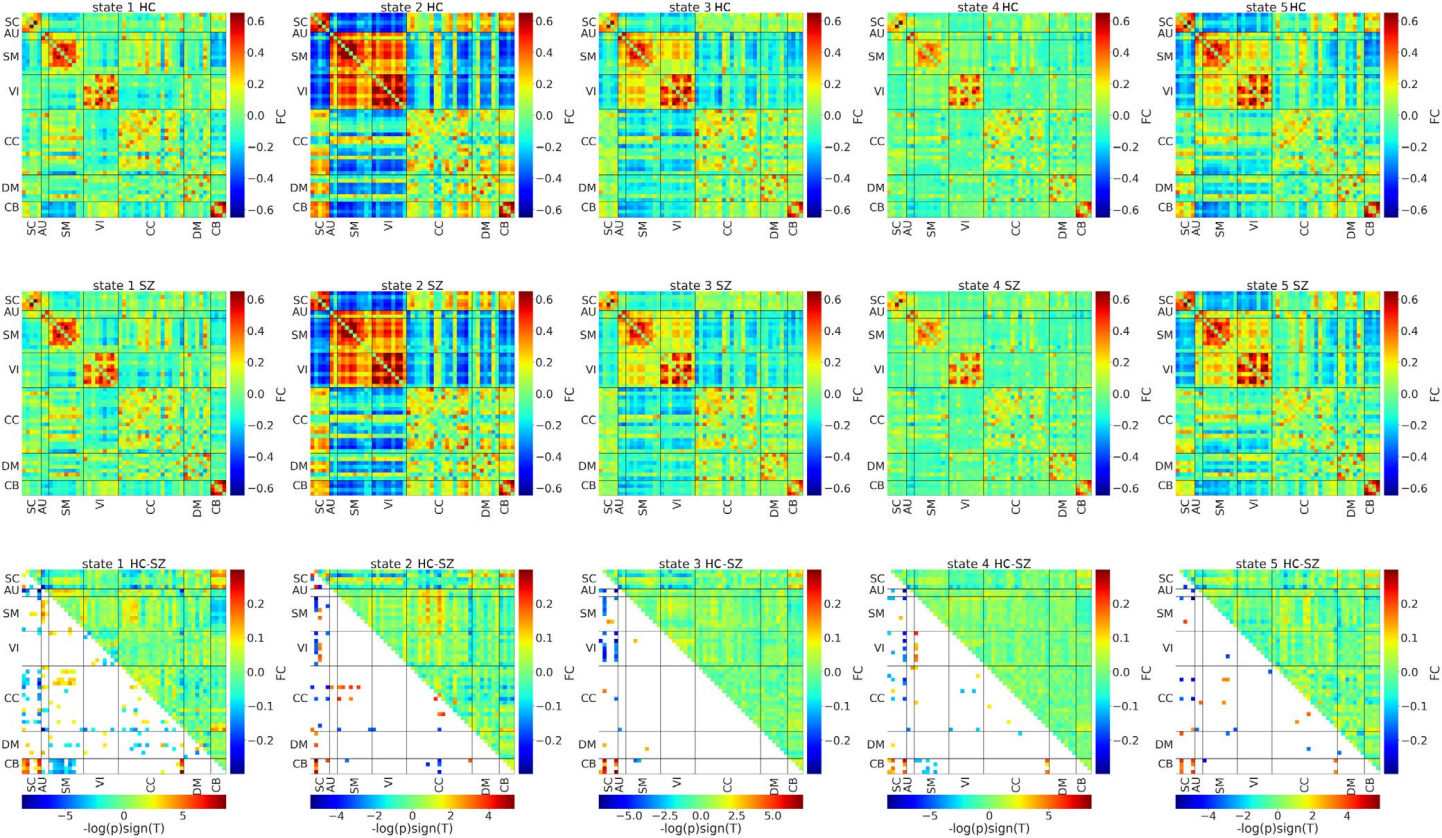
Averaged functional connectivity matrices for control and SZ and group differences. The upper triangle is the differences between averaged correlation matrix of SZ and healthy control (SZ‐HC), and the lower triangle is the result of multiple comparison test. State 3, 5, and 2 exhibit a moderate to high correlation pattern, while states 1 and 4 demonstrate low and weakest connectivity states. Three states (3, 5, and 2) show moderate to high correlation patterns, while states 1 and 4 demonstrate low connectivity. Notably, state 2 exhibits pronounced correlation among AUD, SM, VI, SC, and CB domains, alongside notable antagonism between SC and AU, SM, VI, as well as between AU, SM, VI, and CC, DM, CB. Group difference evaluations reveal varying numbers of significant differences among states, with notable antagonism between cerebellar and sensory intrinsic connectivity networks (ICNs) observed in state 1. Additionally, differences in hyperconnectivity between subcortical and cognitive control ICNs are evident across states, with particular emphasis on the subcortical and cerebellar domains in various states.

Repeated balanced‐sample analyses revealed variable stability of the dynamic path findings. The State 4 sMRI_13–FNC_8 disconnector was recovered in 100% of iterations. High recurrence was also observed for the State 2 FNC_4–FNC_9 and sMRI_7–FNC_13 connectors (86.4% and 82.0%), the State 5 FNC_3–FNC_11 and FNC_3–FNC_6 connectors (85.8% and 77.4%), and the State 1 sMRI_13–FNC_6 connector (76.8%). Other paths showed moderate or limited recurrence, with particularly low stability for many State 2 findings after balancing to 42 participants per group. These findings indicate that some dynamic path alterations are reproducible under balanced sampling, whereas others are more sensitive to state‐specific sample composition.

Figure 10 shows the results of applying path analysis to identify missing and additional links for each state.

**FIGURE 10:**
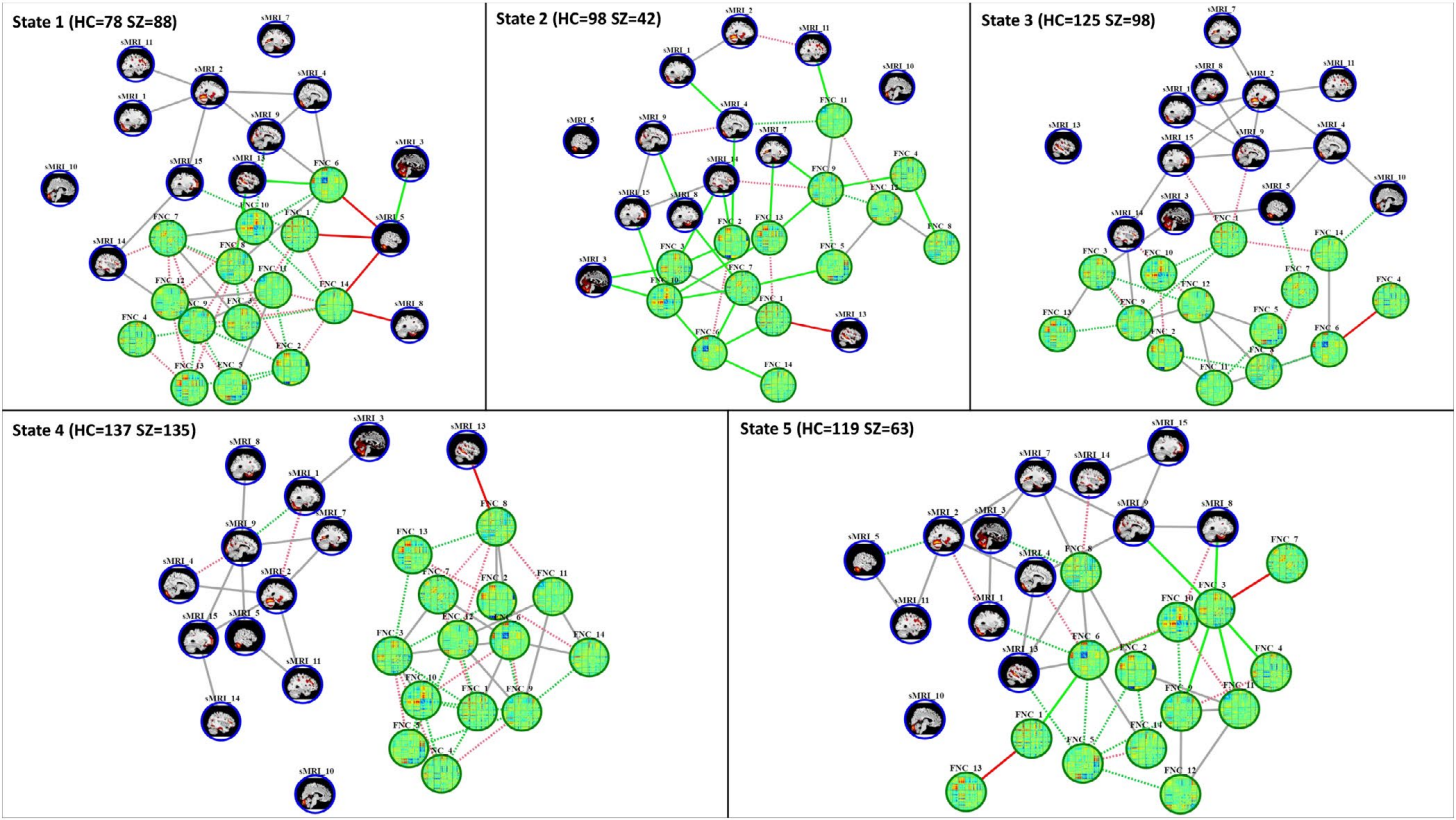
Disconnectors and connectors that were identified in each state. Solid red links trigger disconnection (absence of paths), and solid green links trigger abnormal integration (additional paths) in SZ group with reference to the control group graph. In State 1, multiple multimodal disconnection links emerge involving the cerebellum's gray matter component, showing high connectivity across various intrinsic connectivity network (ICN) pairs and domains. State 2 exhibits both connectors and disconnectors, with notable correlations among sensory motor, visual, and other ICNs. State 3 displays a unimodal disconnection between specific functional network components. State 4 and 5 reveal further multimodal disconnectors between gray matter components and functional network components, highlighting disruptions across sensory, visual, cognitive control, and default mode domains, underscoring the significance of examining multimodal interactions in understanding disorder‐related network disruptions.

## 4 Discussion

In this study, we presented an approach to assess multi‐step graphical paths that span multimodal neuroimaging data. We aimed to identify multimodal path‐based biomarkers for individuals with SZ; however, the proposed method can be applied to any undirected graph estimated from data related to other conditions as well. We proposed a method to estimate static and dynamic multimodal graphs of controls and patients with SZ in a data‐driven manner. We defined data‐driven nodes and edges. We used pICA to define multimodal nodes and sparse Gaussian graphical models to estimate conditional‐dependence edges. For the static analysis, graph sparsity was controlled using a common graphical‐lasso regularization parameter selected according to a prespecified target‐density criterion, thereby applying the same graph‐selection rule to both diagnostic groups. We used GGM to model the human brain as GGMs are powerful tools for expressing statistical relationships between nodes. Bivariate correlation network analysis might result in many spurious edges, as correlation cannot distinguish between direct and indirect associations. In contrast, relationships estimated by GGMs reduce the risk of identifying spurious relationships, as it is a key advantage of partial correlations that reflects conditional independencies (Altenbuchinger et al. 2020).

Path analysis of the revised static multimodal GGMs identified five disconnectors and one connector associated with altered path organization in SZ. Importantly, the path‐based framework distinguished graph differences that modified network reachability from simple edge‐level differences that did not alter the organization of connected components. Across the two graphs, three simple missing and five simple additional edges were observed in addition to the path‐altering edges, illustrating that an edge difference alone does not necessarily imply loss or gain of network‐level reachability.

Most static path‐altering edges occurred within the structural or functional modality; however, one disconnector linked an sMRI component with an sFNC component (sMRI_14–sFNC_14), demonstrating that structure–function relationships also contributed to topology‐level differences between HC and SZ. The graph was constructed within a joint multimodal feature space derived through pICA, allowing structural and functional components to be evaluated simultaneously within the same conditional‐dependence framework. The dynamic analysis identified additional state‐dependent cross‐modal path alterations, suggesting that static and dynamic graph representations provide complementary information about schizophrenia‐related network reorganization.

The number of studies analyzing multimodal probabilistic graphical models is remarkably small as it necessitates broader proficiency in collecting multimodal data, analyzing, modeling probabilistic graphical models, and interpreting the outcome in comparison with unimodal studies. The multimodal study's findings can be complementary to and extend the unimodal analysis. The static and dynamic analyses also differed in the extent of the path alterations detected. The static analysis yielded a relatively small set of path‐altering edges, predominantly within modality but including one cross‐modal disconnector, whereas the dynamic analysis revealed a larger and more heterogeneous set of state‐dependent connectors and disconnectors. These findings emphasize that static and dynamic graph representations provide complementary information. Static connectivity summarizes relationships across the full acquisition, whereas dynamic states may reveal transient alterations that are attenuated when connectivity is averaged over time. The dynamic sensitivity analyses further demonstrated that the stability of these state‐specific findings varies across states and path alterations; therefore, the identified connectors and disconnectors should be interpreted as candidate path‐based markers requiring additional validation.

Table 2 presents a comprehensive summary of the counts for identified connectors and disconnectors across all states in both dynamic and static analyses. Additionally, it provides a tally for multimodal occurrences.

**TABLE 2:** Number of disconnectors and connectors in static and dynamic analysis.

	Disconnectors	Connectors	Multimodal disconnectors	Multimodal connectors
Static analysis	5	1	1	0
State 1	4	3	4	2
State 2	1	23	1	10
State 3	1	0	0	0
State 4	1	0	1	0
State 5	2	7	0	2

Table 3 displays the endpoint of disconnector and connector links in the dynamic analysis corresponding to each state. Table 4 illustrates the sMRI components along with their peak coordinates, while Table 5 displays the FNC components and their associated domains with the highest connectivity.

**TABLE 3:** Links associated with disconnection and abnormal integration.

State	Disconnectors	Connectors
State 1	(sMRI_5, FNC_14) (sMRI_5, FNC_6) (sMRI_5, FNC_1) (sMRI_8, FNC_14)	(sMRI_3, sMRI_5) (sMRI_13, FNC_6) (sMRI_13, FNC_8)
State 2	(sMRI_13, FNC_1)	(FNC_4, FNC_8) (FNC_6, FNC_14) (FNC_7, sMRI_8) (FNC_9, FNC_13) (sMRI_15, FNC_10) (sMRI_7, FNC_13) (sMRI_14, FNC_2) (sMRI_11, FNC_11) (FNC_6, FNC_7) (FNC_6, FNC_10) (FNC_10, FNC_13) (FNC_2, FNC_3) (sMRI_7, FNC_9) (sMRI_8, sMRI_9) (sMRI_3, FNC_10) (FNC_4, FNC_9) (sMRI_4, FNC_2) (sMRI_1, sMRI_4) (FNC_5, FNC_7) (sMRI_14, FNC_3) (FNC_7, FNC_10) (FNC_1, FNC_6) (sMRI_3, FNC_3)
State 3	(FNC_4, FNC_6)	**_**
State 4	(sMRI_13, FNC_8)	**_**
State 5	(FNC_1, FNC_13) (FNC_3, FNC_7)	(FNC_3, FNC_9) (sMRI_8, FNC_3) (FNC_3, FNC_11) (FNC_3, FNC_4) (FNC_1, FNC_6) (sMRI_9, FNC_3) (FNC_3, FNC_6)

**TABLE 4:** sMRI components.

Components	Peak	Coordinate		
		X	Y	Z
sMRI_1	Cerebelum_7b_L	−31.5	−70.2	−52.6
sMRI_2	Cerebelum_6_L	−22.7	−55.5	−19.4
sMRI_3	Cerebellum	−0.5	−56.9	−55.5
sMRI_4	Cerebellum_Crus2_R	12.0	88.6	35.6
sMRI_5	Cerebelum_Crus1_L	−51.4	54.0	−37.8
sMRI_7	Calcarine	25.6	−59.9	7.1
sMRI_8	Temporal_Pole_Mid_L	−28.6	18.3	−40.8
sMRI_9	Calcarine_L	−9.4	−100.4	−10.6
sMRI_10	Cerebelum_Crus2	6.1	−84.2	−31.9
sMRI_11	Frontal_Inf_Tri	33.4	30.1	27.1
sMRI_13	Temporal_Mid	−46.3	−40.7	4.2
sMRI_14	Frontal_Inf_Tri_R	39.3	38.9	5.7
sMRI_15	Frontal_Mid_Orb_L	−30.0	60.3	−6.1

**TABLE 5:** Domain with highest connectivity for FNC components. (DM: Default mode network, VIS: Visual, CC: Cognitive control, SM: Sensorimotor, AU: Auditory, SC: Sub‐cortical, CB: Cerebellar).

Components	Domain connectivity	
	Positive	Negative
FNC_1	AU‐SM, AU‐CC, SM‐SM, SM‐CC, SM‐VI, VI‐CC	SC‐SM, SC‐CC, SM‐CC, SM‐CB, CC‐CC, CC‐CB
FNC_2	AU‐VI, SM‐VI	VI‐CB
FNC_3	AU‐DM, AU‐CC, SM‐CC, SM‐DM, CC‐CC, CC‐DM	CC‐DM, CC‐CB
FNC_4	SC‐CC, SC‐DM, AU‐SM, AU‐VI, AU‐CC, SM‐CB, CC‐CC, CC‐CB	SC‐AU, AU‐CB, VI‐CC, VI‐DM, CC‐CB
FNC_5	VI‐DM	CC‐DM
FNC_6	SC‐VI	VI‐VI
FNC_7	SC‐AU, SC‐SC, AU‐AU, AU‐CC, SM‐SM, VI‐VI, CC‐DM, CC‐CC, DM‐DM, CB‐CB	SC‐CC, AU‐SM, AU‐DM, AU‐CC, CC‐CC
FNC_8	SM‐VI, SM‐CC, VI‐VI, VI‐CC, DM‐CB	SM‐DM, VI‐CC, VI‐DM, CC‐CC, CC‐DM
FNC_9	SC‐CC, SM‐VI, SM‐DM, SM‐CC, VI‐CC, CC‐CB	SC‐VI, SC‐DM, VI‐CB, CC‐CC, CC‐DM, CC‐CB, DM‐CB
FNC_10	AU‐CC, SM‐CC, VI‐CC	VI‐CC, CC‐CC
FNC_11	SC‐AU, AU‐CC, SM‐CB, DM‐CB	SC‐CB, AU‐SM, AU‐DM, SM‐DM, CC‐CB
FNC_12	AU‐DM, SM‐DM, CC‐DM	SM‐CC, CC‐CC, CC‐DM, DM‐CB
FNC_13	SM‐VI, VI‐CC	SM‐CC, VI‐CC, VI‐DM
FNC_14	SC‐AU, SC‐SM, AU‐CC, SM‐CC	SC‐SC, SC‐CC, SC‐DM, SC‐CB, CC‐CC, CC‐CB

In State 1, a multimodal disconnection link emerges between the cerebellum's gray matter component and an FNC component, showcasing high connectivity across the putamen ICN and sensory motor ICNs, caudate ICN and sensory motor ICNs, as well as the sensory motor domain and cognitive control domain and between the cerebellum and the insula. Furthermore, an additional multimodal disconnection link is evident between the cerebellum's gray matter component and an FNC component which exhibits high correlation between sub‐cortical and visual domains and high antagonism within the visual domain. Additionally, there are other multimodal disconnectors between the cerebellum component of gray matter and FNC components which show high connectivity between the sensory domain (postcentral gyrus) and other domains including auditory, cognitive control and visual domains. Another disconnector was observed between the temporal pole component of gray matter and FNC component which shows high connectivity between putamen ICN and sensory motor ICNs, between caudate ICN and sensory motor ICNs, between the sensory motor domain and the cognitive control domain, and between the cerebellum and the insula. State 1 shows a unimodal connector within gray matter components which connects cerebellum components. In addition, a multimodal connector was observed between the temporal middle component of gray matter and FNC component which has high correlation between sub‐cortical and visual domains and high antagonism within the visual domain. Another multimodal connector was identified between the temporal middle component of gray matter and FNC component with high activity across the sensory motor, the visual and the default mode networks.

State 2 shows a multimodal disconnector between the temporal middle component of gray matter and FNC component which has high correlation among sensory motor, visual, and other domains' ICNs. However, state 2 reveals 23 connectors both within and between modalities.

State 3 shows only a unimodal disconnection which is (FNC_4, FNC_6). The FNC_4 shows high activation between the subcortical and the cognitive control, between the subcortical and the default mode network, between the visual domain and cognitive control, and between the auditory domain and all other domains. The FNC_6 reveals higher functional connectivity within the visual domain and between the visual domain and subcortical, cognitive control, and default mode network.

State 4 shows a multimodal disconnector between sMRI_13 which is the middle temporal gyrus and FNC_8 which shows higher correlation between sensory motor (postcentral gyrus, parietal lobule) and the visual (middle temporal gyrus), as well as between the middle temporal gyrus of the visual domain and the frontal gyrus of the cognitive control and the cingulate cortex of the default mode network.

State 5 shows a disconnector between FNC_13 and FNC_1. FNC_13 has higher functional connectivity across the sensory motor, visual, cognitive control, and default mode network domains. The FNC_1 shows high activation across the sensory motor (postcentral gyrus, paracentral lobule), visual (calcarine gyrus, cuneus), and cognitive domains (inferior parietal lobule). In addition, another disconnector was observed between FNC_3 and FNC_7. FNC_3 shows high activation between the sensory and the cognitive control, between the sensory and the default mode, and between the cognitive control and the default mode domains. FNC_7 shows high activation within domains. However, FNC_1 and FNC_3 connected through new additional links in the patient group to other components including FNC_4, FNC_6, FNC_9, and FNC_11 which have higher activation in auditory, visual, subcortical, sensory motor, and cerebellar domains. In addition, FNC_3 connected through an additional multimodal link to sMRI_8 (middle temporal pole) and sMRI_9 (calcarine). Thus, our approach reveals important information about disorder‐related network disruptions that are missed in a focus on single modality.

Several factors should be considered when interpreting the present findings. Head motion was addressed through rigid‐body motion correction and predefined exclusion criteria; however, residual motion effects cannot be completely excluded because a subject‐level motion summary measure such as mean framewise displacement was not included as an additional covariate. Acquisition site, age, and sex were explicitly modeled as nuisance variables in the static multimodal graph analysis, and diagnostic‐group composition did not differ significantly across acquisition sites. Nevertheless, no explicit scanner‐harmonization procedure was applied. Moreover, the state‐specific dynamic graph analyses were not additionally adjusted for acquisition site, and residual site‐related variability may therefore remain in the dynamic findings. Medication exposure, illness duration, and cognitive performance were not included as covariates and may contribute to additional clinical heterogeneity. Future studies should evaluate these potential influences using independent datasets and additional clinical, motion‐related, and site‐specific sensitivity analyses.

Although pICA has previously demonstrated stable performance in simulation studies across variations in signal‐to‐noise ratio, cross‐modal association strength, and model‐order estimation (Liu and Calhoun 2007), these methodological evaluations do not establish the reproducibility of the specific components identified in the present dataset. In the revised static analysis, a common graphical‐lasso regularization parameter was selected using a prespecified target‐density criterion applied identically to the HC and SZ groups. This procedure provides a consistent rule for graph estimation but does not constitute a split‐half, bootstrap, or independent‐sample reproducibility analysis of either the pICA decomposition or the resulting static path findings. Repeated balanced‐sample analyses were used to assess sensitivity of the dynamic path findings to unequal state representation, but this analysis addresses a distinct source of sampling variability. Accordingly, the stability of individual pICA components and path‐based findings should be examined further in independent samples and through dedicated resampling analyses. The connectors and disconnectors identified here should therefore be regarded as candidate path‐based markers rather than established biomarkers.

## 5 Conclusion

In summary, we present a framework for estimating and comparing multimodal brain graphs in both static and dynamic settings, with a particular focus on identifying edges that alter network reachability between HC and SZ groups. By integrating structural and functional imaging features through data fusion and modeling their conditional dependencies using Gaussian graphical models, the proposed approach moves beyond conventional pairwise edge comparisons to characterize alterations in multi‐edge pathways. The static and dynamic analyses provided complementary information, with the dynamic results revealing additional state‐dependent and cross‐modal path alterations. These findings demonstrate the potential utility of path‐based multimodal graph analysis for characterizing network disruption in schizophrenia. The identified connectors and disconnectors should be considered candidate path‐based markers that require further validation in independent and resampled datasets.

## Funding

This work was supported by the National Institutes of Health.

## Conflicts of Interest

The authors declare no conflicts of interest.

## Data Availability

The data that support the findings of this study are available on request from the corresponding author. The data are not publicly available due to privacy or ethical restrictions.
